# Estimated Effectiveness of a Primary Cycle of Protein Recombinant Vaccine NVX-CoV2373 Against COVID-19

**DOI:** 10.1001/jamanetworkopen.2023.36854

**Published:** 2023-10-04

**Authors:** Alberto Mateo-Urdiales, Chiara Sacco, Daniele Petrone, Antonino Bella, Flavia Riccardo, Martina Del Manso, Marco Bressi, Andrea Siddu, Silvio Brusaferro, Anna Teresa Palamara, Giovanni Rezza, Patrizio Pezzotti, Massimo Fabiani

**Affiliations:** 1 Department of Infectious Diseases, Istituto Superiore di Sanità, Rome, Italy; 2 European Programme for Intervention Epidemiology Training, European Centre for Disease Prevention and Control, Stockholm, Sweden; 3 General Directorate of Prevention, Ministero della Salute, Rome, Italy; 4 Office of the President, Istituto Superiore di Sanità, Rome, Italy

## Abstract

**Importance:**

Protein recombinant vaccine NVX-CoV2373 (Novavax) against COVID-19 was authorized for its use in adults in late 2021, but evidence on its estimated effectiveness in a general population is lacking.

**Objective:**

To estimate vaccine effectiveness of a primary cycle with NVX-CoV2373 against SARS-CoV-2 infection and symptomatic COVID-19.

**Design, Setting, and Participants:**

Retrospective cohort study linking data from the national vaccination registry and the COVID-19 surveillance system in Italy during a period of Omicron predominance. All adults starting a primary vaccination with NVX-CoV2373 between February 28 and September 4, 2022, were included, with follow-up ending on September 25, 2022. Data were analyzed in February 2023.

**Exposures:**

Partial (1 dose only) vaccination and full vaccination (2 doses) with NVX-CoV-2373.

**Main Outcomes and Measures:**

Notified SARS-CoV-2 infection and symptomatic COVID-19. Poisson regression models were used to estimate effectiveness against both outcomes. Adjusted estimated vaccine effectiveness was calculated as (1 − incidence rate ratio) × 100.

**Results:**

The study included 20 903 individuals who started the primary cycle during the study period. Median (IQR) age of participants was 52 (39-61) years, 10 794 (51.6%) were female, and 20 592 participants (98.5%) had no factors associated with risk for severe COVID-19. Adjusted estimated vaccine effectiveness against notified SARS-CoV-2 infection in those partially vaccinated with NVX-CoV2373 was 23% (95% CI, 13%-33%) and was 31% (95% CI, 22%-39%) in those fully vaccinated. Estimated vaccine effectiveness against symptomatic COVID-19 was 31% (95% CI, 16%-44%) in those partially vaccinated and 50% (95% CI, 40%-58%) in those fully vaccinated. Estimated effectiveness during the first 4 months after completion of the primary cycle decreased against SARS-CoV-2 infection but remained stable against symptomatic COVID-19.

**Conclusions and Relevance:**

This cohort study found that, in an Omicron-dominant period, protein recombinant vaccine NVX-CoV2373 was associated with protection against SARS-CoV-2 infection and symptomatic COVID-19. The use of this vaccine could remain an important element in reducing the impact of the SARS-CoV-2 pandemic.

## Key Points

**Question:**

What is the estimated effectiveness of a primary cycle with protein recombinant vaccine NVX-CoV2373 in preventing SARS-CoV-2 infection and symptomatic COVID-19 during Omicron-predominant periods?

**Findings:**

In this cohort study of 20 903 adults who started a primary cycle with NVX-CoV2373 in an Omicron-predominant period in Italy, the estimated vaccine effectiveness during the first 4 months after full vaccination was 31% against notified SARS-CoV-2 infection and 50% against symptomatic COVID-19. Estimated effectiveness waned against infection but remained stable against symptomatic COVID-19.

**Meaning:**

These findings suggest that vaccination with NVX-CoV2373 was associated with protection against Omicron infection and symptomatic COVID-19 up to 4 months after completion of the primary cycle.

## Teaser

This cohort study evaluates the estimated effectiveness of a primary vaccination cycle with NVX-CoV2373 against SARS-CoV-2 infection and symptomatic COVID-19 among adults in Italy.

## Introduction

By the end of 2022, the majority of adults in the European Union (EU) had completed the primary cycle of vaccination against COVID-19.^1^ In Italy, the majority of those did so with messenger RNA (mRNA) vaccines (68% with BNT162b2 and 14% with mRNA-1273),^2,3^ approved in December 2020 and January 2021, respectively.^4^ Besides these mRNA vaccines, 5 other vaccines have been approved to date in the EU: 2 adenoviral vector vaccines: ChAdOx1 (AstraZeneca) and Ad26.COV2.S (Janssen); 1 inactivated vaccine: VLA2001 (Valneva); and 2 protein recombinant vaccine: NVX-CoV2373 (Novavax) and VidPrevtyn Beta (Sanofi Pasteur).^4^ Despite the high vaccination coverage, SARS-CoV-2 continues to cause substantial morbidity and mortality in Europe and elsewhere.^5^ Some of the reasons include the emergence of variants of increased transmissibility and the waning of natural and vaccine-acquired immunity over time. A previous study^6^ found that effectiveness of BNT162b2 against symptomatic COVID-19 caused by B.1.1.529 (Omicron) or B.1.617.2 (Delta) decreased from 65% 2 weeks after completing the primary cycle to 8.8% over 25 weeks after the second dose.

In this context, it is important to evaluate whether vaccines using alternative technologies to the ones used by mRNA and adenoviral vaccines are effective in preventing infection and symptomatic COVID-19 against the current dominant variants. In the EU, the European Medicines Agency approved the use of NVX-CoV2373 in those aged 18 years and older in late December 2021. The clinical trial that led to the approval of NVX-CoV2373 found this vaccine to be highly efficacious (over 85%) against B.1.1.7 (Alpha) and previous variants.^7^ Evidence gathered after authorization suggests that NVX-CoV2373 induces similar neutralizing antibody titers as BNT162b2 or mRNA-1273.^8,9,10^ However, to date, we know of no studies based on the general population estimating the absolute or relative effectiveness of NVX-CoV2373. The aim of this study is to estimate the effectiveness of NVX-CoV2373 in preventing SARS-CoV-2 infection and symptomatic COVID-19 in a period dominated by the Omicron variant in Italy, using data from a general population.

## Methods

### Data Sources

We retrieved information on all vaccinations administered to the population from the Italian National Vaccination Registry. This registry is held by the Ministry of Health and collects demographic, vaccine-related, and clinical information at the individual level for each vaccine administration.^11^ Data on SARS-CoV-2 infections and COVID-19 outcomes were obtained through the National Integrated Surveillance system, held by the Italian National Institute of Public Health, which collects information on all notified cases of SARS-CoV-2 infection—confirmed through polymerase chain reaction (PCR) or antigen testing—and follows them up until recovery or death.^12^ We linked deterministically both data sets using an individual unique identifier (tax code). This study followed the Strengthening the Reporting of Observational Studies in Epidemiology (STROBE) reporting guideline.

### Ethics

This study, based on routinely collected data, was not submitted for approval to an ethical committee or an institutional review board as the dissemination of COVID-19 surveillance data was initially authorized by the Italian Decree Law No. 2 on January 14, 2021 (article 3), and subsequently confirmed by Decree Law No. 24 on March 24, 2022 (article 13). Due to the study’s retrospective design and the large size of the population under study, in accordance with the Authorization No. 9 released by the Italian data protection authority on December 15, 2016, individual informed consent was not requested for the conduct of this study.

### Study Design and Population

This is a retrospective cohort study including all adults who received a primary vaccination of NVX-CoV2373 between February 28, 2022, and September 4, 2022. Participants were followed up until September 25, 2022, to ensure that each participant had at least 3 weeks of follow-up. The Omicron variant was dominant throughout the study period. The dominant sublineages were BA.2 from March to May and BA.5 from June to September.^13^

We excluded persons with a previous recorded SARS-CoV-2 infection, those who completed the cycle with a different vaccine from NVX-CoV2373 (heterologous vaccination), and those with missing or inconsistent data. For all included individuals, follow-up started with the first dose administration and finished at the time of diagnosis, if infected, at time of third dose administration, when becoming eligible to receive the third dose (ie, 120 days after completing the primary cycle of 2 doses), or at the end of follow-up (September 25, 2022), whichever came first.^14^

### Outcomes and Exposure

The outcomes of interest were the risk of SARS-CoV-2 infection (confirmed either through PCR or antigen testing) and of symptomatic COVID-19 (an episode of SARS-CoV-2 infection with any associated reported symptom) in those vaccinated with NVX-CoV2373. Risks were analyzed at different periods: during partial vaccination (from day 15 after the first dose until 14 days after the second dose) and during full vaccination (from 15 days after the second dose onwards). We used as our reference period days 3 to 10 after administration of the first dose, assuming that the risk of infection in the first 14 days after first-dose administration was similar to that of unvaccinated individuals, according to information from the trial that led to the authorization of NVX-CoV2373 and using a methodological approach previously reported in other studies^7,15,16^ Days 11 to 14 after the first dose were excluded because, by then, some degree of vaccine-induced immunity may already be present. The first 2 days after first dose administration were also excluded because a deferral bias could affect the likelihood of infection in these days (eg, people with symptoms deferring vaccination).^17^ We did, however, carry out a sensitivity analysis in which our reference period was days 0 to 14 after first dose administration (eTable 1 and eTable 2 in Supplement 1).

### Statistical Analysis

We described the characteristics of the study population using counts with percentages and medians with IQRs for categorical and continuous variables, respectively. Individual data were split in multiple records to account for the calendar week of exposure and the time-varying vaccination status. Then, Poisson regression models, with a robust variance estimator and including time measured in days as offset (difference between the starting date and the ending date of each record-specific interval generated by splitting), were used to estimate incidence rate ratios (IRRs) of both outcomes (SARS-CoV-2 infection or symptomatic COVID-19) for partial and full vaccination. The following potential confounders were included in the model: sex, age group (18-39, 40-59, 60-79 and ≥80 years), macroarea of residence (Nomenclature of Territorial Units for Statistics-1 as per Eurostat^18^) and the macroarea-specific incidence of SARS-CoV-2 infection in the calendar week of exposure. The same model was used to estimate the evolution of protection through time according to each outcome, categorizing the time after full vaccination into 4 time intervals (0-29, 30-59, 60-89, and 90-120 days after completion). IRRs are reported in the form of estimated vaccine effectiveness (1 − IRR) × 100, with 95% CIs. All analyses were carried out using R version 4.1.2 (R Project for Statistical Computing).^19^ We described the methods and presented findings according to the reporting guidelines for observational studies that are based on routinely collected health data. Data were analyzed in February 2023.

## Results

### Study Population

From the 25 547 individuals who started a primary vaccination cycle in Italy from February 28, 2022, to September 4, 2022, we excluded 139 (0.54%) with heterologous vaccination, 4301 (16.8%) who had a previous SARS-CoV-2 infection notified to the surveillance system, 3 persons (0.01%) aged less than 18 years, and 201 (0.79%) who had duplicated records. Table 1 shows the characteristics of the 20 903 persons who were included in the study. The median (IQR) age was 52 (39-61) years, with almost half of participants aged between 40 and 59 years. Participants were more frequently female (10 794 participants [51.6%]), and the vast majority (20 592 participants [98.5%]) had no factors associated with risk for SARS-CoV-2. With regards to the geographical distribution, the most represented macroarea was the northwest (6343 participants [30.3%]) and the least represented was the south and islands (3989 participants [19.1%]). A total of 18 699 participants (89.5%) started the primary cycle before or within March 2022.

**Table 1.** Characteristics of the Study Population

Characteristic	Participants, No. (%) (N = 20 903)
Age, median (IQR), y	52 (39-61)
Age group, y	
18-39	5395 (25.8)
40-59	9552 (45.7)
60-79	5449 (26.1)
≥80	507 (2.4)
Sex	
Female	10 794 (51.6)
Male	10 109 (48.4)
Risk factors	
High clinical risk	192 (0.9)
Healthcare workers	119 (0.6)
No risk factors	20 592 (98.5)
Macroarea of residence	
Center	5426 (26.0)
Northeast	5145 (24.6)
Northwest	6343 (30.3)
South and Islands	3989 (19.1)
First dose administration	
February 2022	957 (4.6)
March 2022	17 742 (84.9)
April 2022	1412 (6.8)
May 2022	446 (2.1)
June 2022	190 (0.9)
July 2022	120 (0.6)
August 2022	36 (0.2)

### Estimated Vaccine Effectiveness

The median (IQR) duration of follow-up was 156 (112-156) days . Rates of SARS-CoV-2 infection were highest in days 3 to 10 after the administration of the first dose (reference period) and lowest after full vaccination (15 days or more after the administration of the second dose) (Table 2). A similar trend was observed for symptomatic COVID-19. Twenty six infections led to hospitalization during the study period; 1 occurred during the reference period, 7 during partial vaccination, and 18 after full vaccination.

**Table 2.** Estimated Vaccine Effectiveness Against Notified SARS-CoV-2 Infection and Symptomatic COVID-19 in Those Starting the Primary Cycle With NVX-CoV2373 From February 28 to September 4, 2022, Italy

Outcome and vaccination status	Person-days	Events, No.	Crude rate (per 100 000 person-days)	Adjusted VE, % (95% CI)
All infections				
3-10 d after first dose	166 074	395	237.8	1 [Reference]
Partial vaccination^a^	510 152	1001	196.2	23 (13-33)
Full vaccination^b^	1 908 025	2811	147.3	31 (22-39)
Symptomatic infection				
3-10 d after first dose	166 074	152	91.5	1 [Reference]
Partial vaccination^a^	510 152	360	70.6	31 (16-44)
Full vaccination^b^	1 908 025	873	45.8	50 (40-58)
Hospitalization				
3-10 d after first dose	166 074	1	0.6	NA
Partial vaccination^a^	510 152	7	1.4	NE
Full vaccination^b^	1 908 025	18	0.9	NE

Abbreviations: NA, not applicable; NE, not estimable because of the low number of events; VE, vaccine effectiveness.

^a^ Fifteen days after first dose to 14 days after second dose.

^b^ Fifteen or more days after second dose.

**Figure 1.**
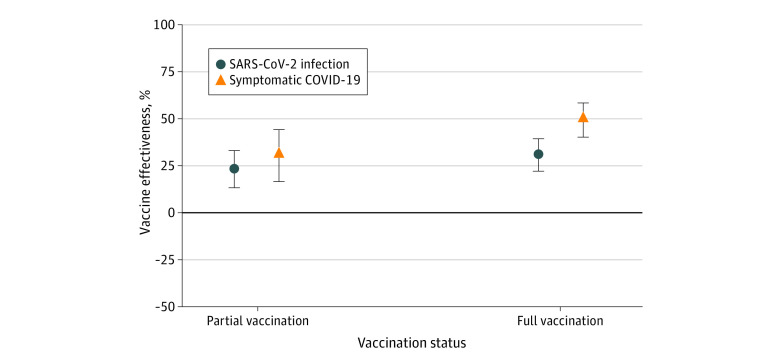
Estimated Vaccine Effectiveness Against Notified SARS-CoV-2 Infection and Symptomatic COVID-19 in Those Vaccinated With NVX-CoV2373

Adjusted estimated vaccine effectiveness against notified SARS-CoV infections in those with partial vaccination (15 days after the first dose to 14 days after the second dose) was 23% (95% CI, 13%-33%), increasing after full vaccination to 31% (95% CI, 22%-39%). Estimated vaccine effectiveness was higher against symptomatic COVID-19, with an estimate of 31% (95% CI, 16%-44%) in those partially vaccinated and of 50% (95% CI, 40%-58%) in those fully vaccinated (Figure 1).

**Figure 2.**
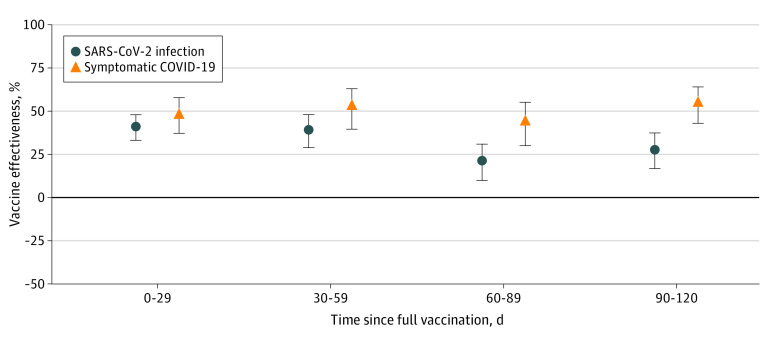
Evolution of Estimated Vaccine Effectiveness Against Notified SARS-CoV-2 Infection and Symptomatic COVID-19 After Completing the Primary Cycle With NVX-CoV2373

Estimated vaccine effectiveness against SARS-CoV-2 infection decreased over time following completion of the primary cycle, from 41% (95% CI, 33%-48%) at 0 to 29 days after full vaccination to 28% (95% CI, 17%-37%) at 90 to 120 days after full vaccination. Estimated vaccine effectiveness against symptomatic COVID-19 remained stable in the first 4 months after full vaccination, from 48% (95% CI, 37%-58%) at 0 to 29 days to 55% (95% CI, 43%-64%) at 90 to 120 days (Table 3 and Figure 2). When including as the reference group the full 0 to 14 days after the first dose administration, lower estimates for both SARS-CoV-2 infection and symptomatic COVID-19 were found, but a similar pattern was observed with higher estimates for vaccine effectiveness against symptomatic COVID-19 than for infection. Equally, we observed in the sensitivity analysis a decrease of estimated effectiveness after full vaccination against SARS-CoV-2 infection but not against symptomatic COVID-19 (eTable 1 and eTable 2 in Supplement 1).

**Table 3.** Vaccine Effectiveness Against Notified SARS-CoV-2 Infection and Symptomatic COVID-19 for NVX-CoV2373 According to Time From Full Vaccination (≥15 Days From Second Dose)

Outcome and vaccination status	Person-days	Events, No.	Crude rate (per 100 000 person-days)	Adjusted VE, % (95% CI)
All infections				
3-10 d after first dose	166 074	395	237.8	1 [Reference]
Full vaccination 0-29 d	504 653	602	119.3	41 (33-48)
Full vaccination 30-59 d	488 225	459	94.0	39 (29-48)
Full vaccination 60-89 d	467 672	983	210.2	21 (10-31)
Full vaccination 90-120 d	447 475	767	171.4	28 (17-37)
Symptomatic infections				
3-10 d after first dose	166 074	152	91.5	1 [Reference]
Full vaccination 0-29 d	504 653	220	43.6	48 (37-58)
Full vaccination 30-59 d	488 225	146	29.9	53 (40-63)
Full vaccination 60-89 d	467 672	296	63.3	44 (30-55)
Full vaccination 90-120 d	447 475	211	47.2	55 (43-64)

## Discussion

In a period when Omicron was the predominant SARS-CoV-2 variant in Italy, our observational study found NVX-CoV2373 vaccination to be associated with effectiveness against SARS-CoV-2 infection and symptomatic COVID-19. Estimated vaccine effectiveness against notified SARS-CoV-2 infection was estimated at 31% for those completing the primary cycle. Estimated effectiveness decreased after full vaccination from 41% during the first month after completion of the primary cycle to 28% 3 to 4 months after full vaccination. Estimated effectiveness against symptomatic COVID-19 was higher, at 50% in those fully vaccinated and without any observed decrease in protection in the first 4 months after the completion of the primary cycle.

The clinical trial that led to the approval of NVX-CoV2373 found estimates of vaccine efficacy of around 95% against infection caused by the original variant of SARS-CoV-2 and of approximately 85% against the B.1.1.7 (Alpha) variant,^7^ similar to the estimates found in the trials that led to the approval of mRNA vaccines (BNT162b2 and mRNA-1273).^20,21^ However, although several observational studies have used clinical data to estimate effectiveness of mRNA vaccines against infection and symptomatic COVID-19, to date there are no studies that we know of estimating the effectiveness of the protein recombinant vaccine NVX-CoV2373. Results from observational studies on mRNA vaccines show that the level of effectiveness depended on the dominant variant. Against the Alpha variant (B.1.1.7), vaccines showed high levels of effectiveness (over 80%) even several weeks after completing the primary cycle.^22^ Against Delta (B.1.617.2), protection against infection remained high during the first weeks after the second dose but waned through time, a phenomenon that was accentuated with the arrival of Omicron (B.1.1.529 and related subvariants).^16^ In a period dominated by Omicron, in Italy, vaccine effectiveness against all notified infections of a primary cycle—in a population where over 90% are vaccinated with either BNT162b2 or mRNA-1273—is estimated at around 30% within 90 days from full vaccination and at 23% 90 to 120 days after full vaccination,^23^ whereas studies in the US found an effectiveness of 30% against BA.5 infection resulting in outpatient visits with a primary cycle of BNT162b2 (less than 6 months from the second dose),^24^ and of 44% against all infections 14 to 90 days after the second dose with mRNA-12373,^25^ similar to the estimates found with NVX-CoV2373 in this study. Equally, a previous study^26^ estimated vaccine effectiveness against symptomatic BA.1 or BA.2 infection at 51.7% with BNT162b2 and at 35.9% with mRNA-1273 after 30 to 90 days after completion of the primary cycle, very similar to our estimates of 53% and 43% against symptomatic COVID-19 at 30 to 59 days and 60 to 89 days after full vaccination, respectively. These epidemiological findings are also in line with published immunological data suggesting similar levels of neutralizing antibodies’ response in individuals vaccinated with NVX-CoV2373, BNT162b2, and mRNA-1273,^8,9^ including during periods of Omicron dominance.^10^

In concordance with previous studies^6,27^ focused on mRNA effectiveness, we observed a decrease in the protection conferred by vaccination against SARS-CoV-2 infection through time. However, observational studies have also reported a decrease in protection against symptomatic COVID-19 after completion of the primary cycle with mRNA vaccines, which was evident within 4 months after completion of the cycle,^26,28^ whereas we did not find any evidence of waning against symptomatic COVID-19. It is unclear whether such discrepancy in the findings is due to a better sustained protection of NVX-CoV2373 compared with mRNA vaccines or whether it is the consequence of methodological and/or symptoms reporting heterogeneity.

This is the first study we know of using observational data to estimate effectiveness of the protein recombinant vaccine NVX-CoV2373, a vaccine authorized for its use in several parts of the world. Estimating clinical effectiveness after authorization is essential for public health authorities to be able to make evidence-based recommendations. Furthermore, this study is based on a period where over 90% of notified SARS-CoV-2 infections were caused by the Omicron variant, which is still the current dominant variant worldwide at the time of writing (February 2023). To assess estimated vaccine effectiveness, we used the first days after the first dose as the control period rather than nonvaccinated individuals. This method helps to avoid biases arising from unknown or unmeasured differences in risk behavior between the vaccinated and the unvaccinated. Such differences are particularly relevant in a context such as Italy at the beginning of 2022, when over 90% of the adult population had completed the primary cycle.^2^ This approach also avoids biases that would arise from a direct comparison between those vaccinated with mRNA vaccines and those vaccinated with NVX-CoV2373, as we do not know what drove some individuals to wait for a protein recombinant vaccine to start primary vaccination, and whether those motivations are associated with risk behaviors, which would make it inappropriate to directly compare them with those initiating a primary cycle with mRNA vaccines in the same period.

### Limitations

This study has some limitations. We only assessed the estimated effectiveness of the primary cycle of vaccination, given that NVX-CoV2373 was not authorized until recently as a booster dose, and we stopped follow-up after 4 months, when booster doses are recommended. Therefore, the public health implications of these findings are limited in contexts where the majority of the adult population have already received more than 2 vaccine doses. Also, due to low numbers, we could not assess estimated effectiveness against hospitalization or death or stratify our results by age group. One limitation inherent to the method used to estimate effectiveness is the possibility that individuals increase risky behaviors after full immunization, which has been described as the Peltzman Effect.^29^ If so, estimates of vaccine effectiveness may be underestimated. By contrast, depletion of susceptibles might have introduced a bias toward overestimation in our estimates because vaccinated persons at higher risk were more likely to be infected during the reference period (3 to 10 days after the first dose) and therefore not considered to estimate the hazard of the outcome at later intervals after vaccination. However, our analysis was adjusted to account for several differences in baseline characteristics among individuals included in the analysis at different intervals, although a residual bias due to uncontrolled confounders might remain. Another limitation is that due to the high levels of viral transmission during the period under study (over 9 million notified infections to the surveillance system) and the increasing use of self-diagnosis through at-home testing, it is likely that an important percentage of SARS-CoV-2 infections were not notified to the surveillance system and therefore not captured in the analysis. This underascertainment could be a source of bias if it was not homogeneous during the study period, as the majority of individuals included in the study received the first dose within March 2022. Additionally, our study population consisted almost exclusively of individuals under 80 years of age and without any high-risk conditions; thus, our estimates of vaccine effectiveness cannot be generalized to the whole population.

## Conclusions

Overall, vaccination with 2 doses of NVX-CoV2373 was associated with protection against SARS-CoV-2 infection and against symptomatic COVID-19 during an Omicron-predominant period in Italy. Estimated effectiveness waned during the first 4 months after completion of the primary cycle against SARS-CoV-2 infection but remained stable against symptomatic COVID-19.

## Notes

Supplement 1. **eTable 1. **Vaccine Effectiveness Against Notified SARS-CoV-2 Infection and Symptomatic COVID-19 in Those Starting the Primary Cycle With Novavax (NVX-CoV2373) From February 28 to September 4, 2022, Italy **eTable 2. **Vaccine Effectiveness Against Notified SARS-CoV-2 Infection and Symptomatic COVID-19 for Novavax (NVX-CoV2373) According to Time From Full Vaccination (≥15 Days From Second Dose)

Supplement 2.
Nonauthor Collaborators

Supplement 3.
Data Sharing Statement
