# Supplementary material for: Estimated Effectiveness of a Primary Cycle of Protein Recombinant Vaccine NVX-CoV2373 Against COVID-19
Source: JAMA Netw Open. 2023 Oct 4;6(10):e2336854. doi: 10.1001/jamanetworkopen.2023.36854 (PMC10551773; doi:10.1001/jamanetworkopen.2023.36854)
Supplement: Supplement 1. — eTable 1. Vaccine Effectiveness Against Notified SARS-CoV-2 Infection and Symptomatic COVID-19 in Those Starting the Primary Cycle With Novavax (NVX-CoV2373) From February 28 to September 4, 2022, Italy eTable 2. Vaccine Effectiveness Against Notified SARS-CoV-2 Infection and Symptomatic COVID-19 for Novavax (NVX-CoV2373) According to Time From Full Vaccination (≥15 Days From Second Dose) [file jamanetwopen-e2336854-s001.pdf]

Supplemental Online Content

Mateo-Urdiales A, Sacco C, Petrone D, et al; Italian National COVID-19 Integrated Surveillance System and the Italian COVID-19 vaccines registry. Estimated effectiveness of a primary cycle of protein recombinant vaccine NVX-CoV2373 against COVID-19. *JAMA Netw Open*. 2023;6(10):e2336854. doi:10.1001/jamanetworkopen.2023.36854

eTable 1. Vaccine Effectiveness Against Notified SARS-CoV-2 Infection and Symptomatic COVID-19 in Those Starting the Primary Cycle With Novavax (NVX-CoV2373) From February 28 to September 4, 2022, Italy

eTable 2. Vaccine Effectiveness Against Notified SARS-CoV-2 Infection and Symptomatic COVID-19 for Novavax (NVX-CoV2373) According to Time From Full Vaccination (≥15 Days From Second Dose)

© 2023 Mateo-Urdiales A et al. *JAMA Network Open.*

Results from sensitivity analysis where the reference period is days 0-14 post first dose administration.

eTable 1. Vaccine Effectiveness Against Notified SARS-CoV-2 Infection and Symptomatic COVID-19 in Those Starting the Primary Cycle With Novavax (NVX-CoV2373) From February 28 to September 4, 2022, Italy

Outcome	Vaccination status	Person-days	Events	Crude rate (per 100 000 person-days)	VE (95% CI)
**All infections**	0-14 days post first dose	310,650	622	200.2	Reference
	Partial vaccination*	510,152	1,001	196.2	8% (-3%-17%)
	Full vaccination**	1,908,025	2,811	147.3	17% (8%-25%)
**Symptomatic infection**	0-14 days post first dose	310,650	225	72.4	Reference
	Partial vaccination*	510,152	360	70.6	12% (-4%-26%)
	Full vaccination**	1,908,025	873	45.8	35% (25%-44%)
**Hospitalisation**	0-14 days post first dose	310,650	3	1.0	-
	Partial vaccination*	510,152	7	1.4	-
	Full vaccination**	1,908,025	18	0.9	-

IRR = Incidence Rate Ratio.
VE = Vaccine Effectiveness.
*15 days post first dose to 14 days post second dose
**15+ days post second dose

© 2023 Mateo-Urdiales A et al. *JAMA Network Open.*

eTable 2. Vaccine Effectiveness Against Notified SARS-CoV-2 Infection and Symptomatic COVID-19 for Novavax (NVX-CoV2373) According to Time From Full Vaccination (≥15 Days From Second Dose)

Outcome	Vaccination status	Person-days	Events	Crude rate (per 100 000 person-days)	VE (95% CI)
All infections	0-14 days post first dose	310,650	622	200.2	Reference
	Full vaccination 0-29 days	504,653	602	119.3	29% (17%-39%)
	Full vaccination 30-59 days	488,225	459	94.0	26% (12%-38%)
	Full vaccination 60-89 days	467,672	983	210.2	5% (-15%-22%)
	Full vaccination 90-120 days	447,475	767	171.4	14% (-8%-31%)
Symptomatic infections	0-14 days post first dose	310,650	225	72.4	Reference
	Full vaccination 0-29 days	504,653	220	43.6	34% (23%-43%)
	Full vaccination 30-59 days	488,225	146	29.9	39% (28%-49%)
	Full vaccination 60-89 days	467,672	296	63.3	28% (13%-40%)
	Full vaccination 90-120 days	447,475	211	47.2	42% (28%-54%)

© 2023 Mateo-Urdiales A et al. *JAMA Network Open.*
