# Supplementary material for: Estimated Effectiveness of a Primary Cycle of Protein Recombinant Vaccine NVX-CoV2373 Against COVID-19
Source: JAMA Netw Open. 2023 Oct 4;6(10):e2336854. doi: 10.1001/jamanetworkopen.2023.36854 (PMC10551773; doi:10.1001/jamanetworkopen.2023.36854)
Supplement: Supplement 2. — Nonauthor Collaborators [file jamanetwopen-e2336854-s002.pdf]

Supplemental Online Content: Nonauthor Collaborators
*First name, last name, and suffix (if applicable) are required and will appear in PubMed.

***Group Name(s): Italian National COVID-19 Integrated Surveillance System and the Italian COVID-19 vaccines registry.**

***First Name and Middle Initial(s)**	***Last Name**	***Suffix (eg, Jr, III)**	Academic Degrees	Institution	Location (city, state/province, country)	Role or Contribution, eg, chair, principal investigator	Group (if more than 1 Group listed in the byline) and/or Subgroup (eg, Steering Committee)
Stefano	Boros						
Fortunato (Paolo)	D'Ancona						
Corrado	Di Benedetto						
Antonietta	Filia						
Maria Cristina	Rota						
Marco	Tallon						
Maria Fenicia	Vescio						
Antonia	Petrucci						
Michele	La Bianca						
Anna Domenica	Mignuoli						
Pietro	Buono						
Erika	Massimiliani						
Fabio	Barbone						
Francesco	Vario						
Camilla	Sticchi						
Danilo	Cereda						
Marco	Pompili						
Francesco	Sforza						
Pierpaolo	Bertoli						
Pier Paolo	Benetollo						
Chiara	Pasqualini						
Lucia	Cisceglia						
Maria Antonietta	Palmas						
Sebastiano Pollina	Addario						
Emanuela	Balocchini						
Anna	Tosti						
Mauro	Ruffier						
Filippo	Da Re						
Serena	Battilomo						
Valeria	Proietti						

Supplemental Online Content: Nonauthor Collaborators
*First name, last name, and suffix (if applicable) are required and will appear in PubMed.

***First Name and Middle Initial(s)**	***Last Name**	***Suffix (eg, Jr, III)**	Academic Degrees	Institution	Location (city, state/province, country)	Role or Contribution, eg, chair, principal investigator	Group (if more than 1 Group listed in the byline) and/or Subgroup (eg, Steering Committee)
Camillo	Odio						
Michele	Recine						
Innocenza	Ruberto						
Salvatore	Ascione						
Massimo	Bisogno						
Gandolfo	Miserendino						
Massimiliano	Navacchia						
Beatrice	Del Frate						
Emanuela	Cau						
Diego	Baiocchi						
Danilo	Fusco						
Domenico	Gallo						
Maria Rosa	Marchetti						
Diego	Conforti						
Carlo	Trentini						
Antonino	Ruggeri						
Concetta	Ladalardo						
Nehludoff	Albano						
Marco	Corona						
Paolo	Lombardi						
Massimo	Iacono						
Paolo Bruno	Angori						
Andrea	Belardinelli						
Milena	Solfiti						
Stefano	Fioraso						
Chiara	Poma						
Nadia	Raccanello						
