# Supplementary material for: Estimated Effectiveness of a Primary Cycle of Protein Recombinant Vaccine NVX-CoV2373 Against COVID-19
Source: JAMA Netw Open. 2023 Oct 4;6(10):e2336854. doi: 10.1001/jamanetworkopen.2023.36854 (PMC10551773; doi:10.1001/jamanetworkopen.2023.36854)
Supplement: Supplement 3. — Data Sharing Statement [file jamanetwopen-e2336854-s003.pdf]

Data Sharing Statement

Mateo-Urdiales. Estimated Effectiveness of a Primary Cycle of Protein Recombinant Vaccine NVX-CoV2373 Against COVID-19. *JAMA Netw Open*. Published October 04, 2023. doi:10.1001/jamanetworkopen.2023.36854

Data
Data available: No
